# Assessing the association between area deprivation index on COVID-19 prevalence: a contrast between rural and urban U.S. jurisdictions

**DOI:** 10.3934/publichealth.2021042

**Published:** 2021-07-22

**Authors:** Christopher Kitchen, Elham Hatef, Hsien Yen Chang, Jonathan P Weiner, Hadi Kharrazi

**Affiliations:** 1 Center for Population Health IT, Department of Health Policy and Management, Johns Hopkins Bloomberg School of Public Health, Baltimore, MD, USA; 2 Division of Health Sciences Informatics, Johns Hopkins School of Medicine, Baltimore, MD, USA

**Keywords:** COVID-19, area deprivation index, rural health, social determinants of health, health disparities research

## Abstract

**Background:**

The COVID-19 pandemic has impacted communities differentially, with poorer and minority populations being more adversely affected. Prior rural health research suggests such disparities may be exacerbated during the pandemic and in remote parts of the U.S.

**Objectives:**

To understand the spread and impact of COVID-19 across the U.S., county level data for confirmed cases of COVID-19 were examined by Area Deprivation Index (ADI) and Metropolitan vs. Nonmetropolitan designations from the National Center for Health Statistics (NCHS). These designations were the basis for making comparisons between Urban and Rural jurisdictions.

**Method:**

Kendall's Tau-B was used to compare effect sizes between jurisdictions on select ADI composites and well researched social determinants of health (SDH). Spearman coefficients and stratified Poisson modeling was used to explore the association between ADI and COVID-19 prevalence in the context of county designation.

**Results:**

Results show that the relationship between area deprivation and COVID-19 prevalence was positive and higher for rural counties, when compared to urban ones. Family income, property value and educational attainment were among the ADI component measures most correlated with prevalence, but this too differed between county type.

**Conclusions:**

Though most Americans live in Metropolitan Areas, rural communities were found to be associated with a stronger relationship between deprivation and COVID-19 prevalence. Models predicting COVID-19 prevalence by ADI and county type reinforced this observation and may inform health policy decisions.

## 1. Introduction

The 2019–2021 coronavirus pandemic has underscored many of public health disparities in the United States. Minority communities and people living in poverty account for disproportionately more COVID-19 cases and fatalities [1],[2]. The same communities may be inherently more vulnerable, due to underlying health conditions, poverty and lack of access to care [3]–[5]. Comparatively, less attention has been given to the spread of COVID-19 in rural communities, even though recent evidence suggests a rapid spread in rural areas [6].

Greater prevalence of chronic disease and remoteness of rural areas are cause for concern, even though they make up only a fraction of total COVID-19 cases in the U.S. [7],[8]. Rural communities are more vulnerable to economic hardship, have worse healthcare access, health literacy and outcomes [9]–[12]. By extension, we may expect worse outcomes for more impoverished rural jurisdictions during the pandemic [11],[12].

Past health disparities research has established a relationship between poor health outcomes and low socioeconomic status, often taken as a ranked measure of geographic area deprivation index, or ADI [13],[14]. Few researchers have made use of ADI when evaluating COVID-19 prevalence across U.S. geographies, but early evidence seems to confirm a general positive relationship between deprivation and prevalence exists [15],[16]. The ADI also permits inspection of its individual components to better understand nuanced or subtle population effects of social determinants of health (SDH), at the county level [17]. Other models, such as the social vulnerability index (SVI), may not be as readily amenable to the county level geography [18]. Proper disease management and policy efforts must understand these contrasts and public health needs to properly combat the spread of COVID-19 [19].

ADI is an important tool for this discovery as it is publicly available and identifies which communities are at risk for poor health outcomes (e.g. mortality, hospitalization, emergency care, etc.). Effective policy could be validated and informed by such an index. ADI is used in this analysis as a predictor for COVID-19 prevalence that permits contrast between diverse communities. Our hypothesis is that ADI and its components are predictive of COVID-19 prevalence and that this correspondence is differentiated at least partially by county type.

## 2. Materials and methods

### 2.1. Data sources

Current estimates for COVID-19 cases were obtained from the JHU CSSE Coronavirus tracking project [20],[21]. This data repository contains county level time series data for confirmed cases reported to the U.S. Centers for Disease Control and Prevention (CDC) dating back to January 22^nd^, 2020 and commonly used by population health researchers for modeling COVID-19 spread [22]–[24]. We selected cumulative COVID-19 case estimates as of August 20^th^, 2020 for analysis. This was the latest data we had retrieved before a resurgence in cases thru Winter 2021, which may represent the start of a distinct, seasonal phase in the ongoing pandemic. Population by race/ethnicity, and gender per county were based on 2019 estimates from the 2010 U.S. Census [25],[26]. Case prevalence was calculated as a count of confirmed cases per 100k persons in each county. County data were linked across sources using their unique Federal Information Processing System (FIPS) geocodes.

### 2.2. Ethical issues

COVID-19 prevalence and population characteristics are made publicly available by the US CDC and US Census Bureau respectively. No personally identifiable or protected health information was included as part of this research and no attempt was made to associate cases to either identifying information or protected health records. This analysis was therefore exempt from institutional review and approval.

### 2.3. Area Deprivation Index

We constructed county level ADI by weighting 17 widely used measures in population health literature for poverty, income, and education [13],[27],[28]. The 5-year estimates of 2018 American Community Survey (ACS) data were used for calculating ADI and each of the composite measures, using an approach as described by Singh et al. [13],[26],[28]. Higher raw ADI corresponds to more deprivation and therefore lower socioeconomic status (SES). A high ADI national percentile rank corresponds to high raw ADI and more deprivation. We made use of national rank ADI for modeling of COVID-19 prevalence.

### 2.4. Urban vs. rural designation

We classified 3,142 counties across the U.S. as “urban” or “rural” and stratified the relationship between prevalence and ADI accordingly. It was necessary to rely on a classification scheme developed for the county level geography. The National Center for Health Statistics (NCHS) developed such a mechanism for classifying rural and urbanized areas in 2001 for the accurate assessment and measurement of health differences between residents [29]–[30]. The 2013 NCHS Urbanization scheme defines Metropolitan Statistical Areas (MSA) as at least 50,000 residents with an urban nucleus of at least 1,000 persons per square mile. Urban counties possess an urbanized core or are surrounding counties with at least 500 people per square mile included in the MSA. Nonmetropolitan counties (hereafter, “rural”) are micropolitan or noncore geographies of fewer than 50,000 residents.

### 2.5. Statistical tests

Descriptive statistics for population, population density, ADI, ADI components, Census variables and COVID-19 case-mortality figures were tabulated across county type. Effect sizes for each comparison were estimated using Kendall's tau and considered statistically significant at a p < 0.001 level. Additional county-level social determinants of health (SDH) variables included percent male, percent non-Caucasian minority and percent aged 65 years or older. A subset of SDH variables are presented in this work to reduce redundancy of ADI measures, while illustrating resident demographics and domains of the ADI.

Spearman rank correlation coefficients were calculated for ADI national rank, ADI components and prevalence estimates and for each county type. These correlation statistics were summarized as correlation matrices for inspection. All underlying rho coefficients and p-values were calculated, but only a subset presented as part of the results.

Finally, five models using logarithmic link functions were fitted to explore an effect of county type (i.e., urban vs. rural) on the relationship between ADI and COVID-19 prevalence. A base comparison model is defined as a mapping of ADI national rank to cumulative COVID-19 prevalence. Pairs of test models reflect stratification based on county type. Model 2 and 3 fitted national rank ADI to COVID-19 Prevalence for Urban and Rural jurisdictions respectively. Models 4 and 5 fitted constituent variables of ADI to COVID-19 Prevalence with respect to county type. For each model, we compared relative residual deviance and McFadden R^2^ as an OLS analogue for deviance explained [31],[32]. This permitted comparison of either constituent model (models 4 and 5) with their corresponding ADI base model (models 2 and 3). Inspection of model effect sizes allowed us to interpret which features of ADI contributed most to differential performance by county type. This was summarized as a variable importance plot ranking the absolute t-values obtained from inputs of models 4 and 5.

## 3. Results

### 3.1. Characteristics of urban vs. rural

Table 1 reflects common SDH, including household income (in USD), percent of families below poverty, percent of households without vehicles and percent of households with more than one person per bedroom. Rural counties were found to have significantly worse outcomes, including median family income (mean = $59,097) and percent of residents under 150% of poverty (mean = 28%). They were also characteristically more male (mean = 50.4%), had fewer non-Caucasian residents (mean = 15.4%) and more residents aged 65 or older (mean = 17.1%). No significant difference was found in percent of households with more than one occupant per bedroom (mean = 2.5%), percent unemployed (mean = 5.8%) or percent single parent households (mean = 34.1%). Rural counties had significantly fewer COVID-19 average cases, cases per capita and deaths as of August 20, 2020.

**Table 1.** Population characteristics for ADI, ADI components, SDH and cumulative COVID-19 case, prevalence and mortality.

Feature	Rural Mean (SD) or N (%)	Urban Mean (SD) or N (%)	Effect Size τ (p)	All Counties Mean (SD) or N (%)
Count of Counties	1,976 (62.9%)	1,166 (37.1%)	-	3,142 (100%)
Characteristics				
Total Population (1,000)	46,063 (14%)	282,176 (86%)	-	328,240 (100%)
Mean Population (1,000)	23.3 (22.2)	242.0 (518.5)	0.476 *	104.5 (333.4)
Area (Sq Miles)	1,2789 (4,303)	901 (1,652)	-0.107 *	1,138 (3,562)
Density	42.9 (95)	625.7 (2,7917)	0.493 *	259.3 (1,725)
ADI Variables				
National Rank ADI	56.6 (26.5)	37 (26.7)	-0.276 *	49.3 (28.2)
Median Family Income (1,000 USD)	59.1 (12.1)	72.5 (17.9)	0.323 *	64.1 (15.9)
Median Mortgage (1,000 USD)	1.1 (212.4)	1.4 (411.1)	0.401 *	1.2 (337.5)
Median Rent (1,000 USD)	672.5 (137.1)	899.9 (248.1)	0.448 *	756.9 (216.1)
Median House Value (1,000 USD)	122.0 (65.5)	190.1 (108.5)	0.381 *	147.2 (90.3)
% Families in Poverty	12 (6.1)	9.9 (4.4)	-0.136 *	11.2 (5.7)
% Owner Occupied Housing	72.4 (7.1)	69.8 (9.7)	-0.097 *	71.4 (8.3)
Ratio Earning <$10k to >$50k	2.7 (0.6)	2.4 (0.6)	-0.23 *	2.6 (0.7)
% Under 150pct Poverty	28 (8.8)	22.7 (7.5)	-0.227 *	26 (8.7)
% Single Parent Households	34.1 (10.4)	33.4 (8.3)	-0.024 (0.09)	33.8 (9.6)
% No Vehicle Households	6.4 (4.5)	6.3 (4.5)	-0.034 (0.02)	6.4 (4.5)
% with White Collar Jobs	30.2 (5.6)	34.7 (7.3)	0.266 *	31.9 (6.7)
% Unemployed	5.8 (3.3)	5.7 (2)	0.015 (0.31)	5.8 (2.8)
% ≥ HS Education	85.6 (6.7)	88.2 (5.2)	0.153 *	86.6 (6.3)
% < 9th Grade Education	5.5 (4)	4.3 (2.8)	-0.136 *	5 (3.6)
% > 1 Person per Room Households	2.5 (2.8)	2.4 (1.8)	0.019 (0.18)	2.4 (2.4)
Other SDH				
% Male	50.4 (2.7)	49.5 (1.7)	-0.184 *	50.1 (2.4)
% Non-Caucasian Race	15.4 (17.5)	19.6 (15.4)	0.189 *	16.9 (16.9)
% 65yoa or Older	17.1 (3.9)	14.4 (3.5)	-0.296 *	16.1 (4)
COVID-19 Prevalence				
Confirmed Cases	296.3 (446.4)	4,205 (12,202)	0.439 *	1,747 (7,675)
Cases per 100,000	1,199 (1,264)	1,391.6 (1,072)	0.156 *	1,271 (1,200)
Deaths	6.7 (14.2)	136.8 (487.4)	0.418 *	55 (303.6)

Note: *p-values are significant at the p < 0.001 level. Values reflect CSSE estimates as of 8/20/2020.

### 3.2. Correlation between prevalence and ADI by county type

**Figure 1.**
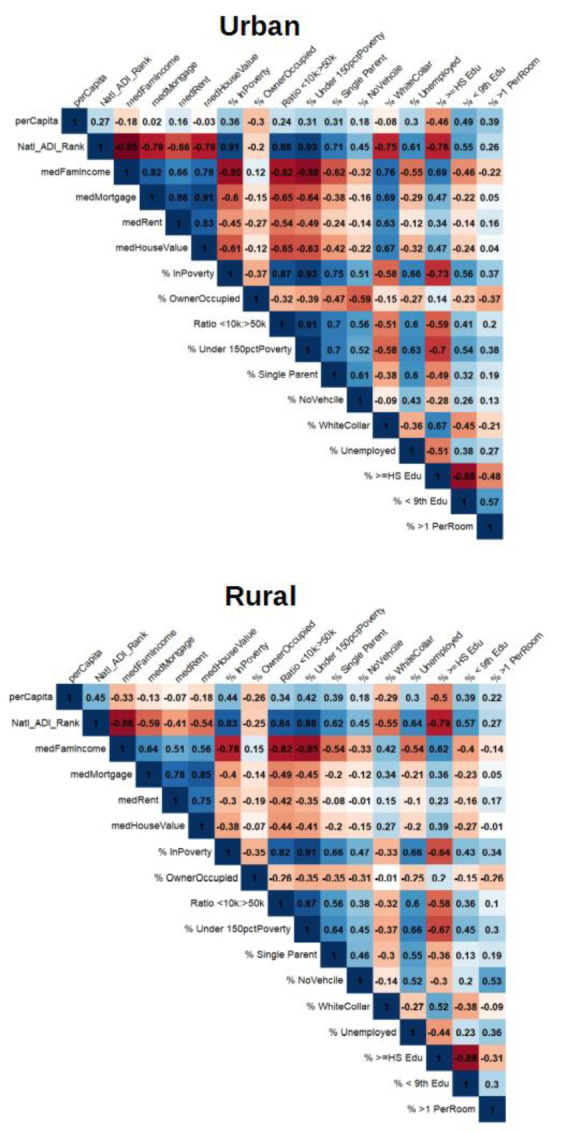
Correlation matrices for COVID-19 prevalence and ADI components across county type.

COVID-19 prevalence was higher in urban counties, but less correlated to national rank ADI when compared to rural (ρ = 0.27; 0.45, respectively) (Figure 1). Prevalence for urban counties was also less strongly correlated with family income (ρ = −0.18; −0.33), percent of households under 150% of poverty (ρ = 0.31; 0.42), and percent of residents with a white-collar job (ρ = −0.08; −0.29). In urban counties, prevalence was more correlated with % of residents with less than 9^th^ grade education (ρ = 0.49; 0.39, respectively) and percent of households with more than one person per bedroom (ρ = 0.39; 0.22, respectively). The aforementioned observations were each significant at the p < 0.001 level.

### 3.3. Modeling prevalence by ADI and county type

The base model for overall county level prevalence as a function of ADI (Model 1) yielded a large total residual deviance and only around 16% of deviance explained (Table 2). The parameter estimate for ADI was significant, but a unit increase in ADI rank was only associated with 1.2% change in prevalence (Table 3). ADI within urban jurisdictions (Model 2) was less predictive of prevalence (McFadden R^2^ = 0.132) but had better set of deviance residuals than did the rural comparison, Model 3. The estimated change in prevalence from a unit increase in ADI was around 0.9% for urban counties, and more than double (2%) for rural. Models 4 and 5 obtained roughly equal McFadden R^2^ values for urban and rural jurisdictions (0.371 and 0.386). Compared to their simpler counterparts (Models 2 and 3), both model 4 and 5 had substantial improvements in deviance explained but median deviance residual remained unchanged for rural counties.

**Table 2.** Model performance obtained by COVID-19 prevalence predicted by ADI, ADI components and county type.

Model	County Subset	Predictor(s)	Total Residual Deviance	Residual DF	Mean Deviance Residual	Median Deviance Residual	IQR Deviance Residual	McFadden R2
M1	All	ADI	2354982	3140	−3.42	−0.20	−23.1–13.1	0.16
M2	Urban	ADI	659035.9	1164	−2.46	−0.13	−19.5–11.7	0.13
M3	Rural	ADI	1466136	1974	−3.44	−0.21	−22.3–11.9	0.28
M4	Urban	ADI Components	475069	1150	−1.86	−0.10	−15.2–8.6	0.37
M5	Rural	ADI Components	1243785	1960	−3.22	−0.21	−20.0–10.0	0.39

**Table 3.** Model coefficients and effect sizes for COVID-19 prevalence by ADI, ADI components and county type.

Features	Coefficient Estimate	2.50%	97.50%	z value	y-change	y-change/%
M1: All Prevalence~ADI						
(Intercept)	6.48	6.48	6.48	5434.5	-	0
National Rank ADI	0.01	0.01	0.01	669.6	8.08	1.24
M2: Urban Prevalence~ADI						
(Intercept)	6.87	6.87	6.87	4670.6	-	0
National Rank ADI	0.01	0.01	0.01	321.7	8.84	0.92
M3: Rural Prevalence~ADI						
(Intercept)	5.81	5.83	5.84	2871.4	-	0
National Rank ADI	0.02	0.02	0.02	716.3	6.85	2.01
M4: Urban Prevalence~ADI Components						
(Intercept)	13.22	13.12	13.32	250.2	-	0
Median Family Income	<0.01	<0.01	<0.01	30.5	2.84	<0.01
Median Mortgage	<0.01	<0.01	<0.01	43.8	164.49	0.03
Median Rent	<0.01	<0.01	<0.01	133.8	609.15	0.11
Median House Value	<0.01	<0.01	<0.01	−155.6	−1.67	<0.01
% Families in Poverty	−1.58	−1.67	−1.49	−34	−437509.1	−79.38
% Owner Occupied Housing	−0.76	−0.78	−0.73	−59.2	−292992.3	−53.16
Ratio Earning <$10k to >$50k	0.08	0.07	0.09	25	46339.70	8.41
% Under 150pct Poverty	0.74	0.67	0.82	19.3	604855.63	109.74
% Single Parent Households	1.11	1.08	1.14	67.1	1122006.1	203.57
% No Vehicle Households	−0.59	−0.64	−0.55	−25.2	−246464.3	−44.2
% with White Collar Jobs	0.88	0.83	0.92	38.7	771882.91	140.04
% Unemployed	0.70	0.60	0.81	13.3	561781.79	101.92
% ≥HS Education	−8.49	−8.59	−8.40	−177	−551061.6	−99.98
% <9^th^ Grade Education	−4.04	−4.18	−3.90	−55.6	−541456.2	−98.24
% >1 Person per Bedroom Households	1.54	1.41	1.67	22.8	2022451.8	366.94
M5: Rural Prevalence~ADI Components						
(Intercept)	12.15	12.10	12.20	445.7	-	0
Median Family Income	<0.01	<0.01	<0.01	107.3	2.87	<0.01
Median Mortgage	<0.01	<0.01	<0.01	11.1	14.72	0.01
Median Rent	<0.01	<0.01	<0.01	48.4	77.39	0.04
Median House Value	<0.01	<0.01	<0.01	−52.7	−0.22	<0.01
% Families in Poverty	1.57	1.52	1.62	65.1	716431.05	379.39
% Owner Occupied Housing	−0.91	−0.93	−0.89	−92.6	−112826.4	−59.75
Ratio Earning <$10k to >$50k	−0.06	−0.06	−0.05	−22.4	−10059.14	−5.33
% Under 150pct Poverty	0.77	0.73	0.82	33	220215.32	116.62
% Single Parent Households	2.15	2.13	2.17	243	1433100.1	758.91
% No Vehicle Households	−2.52	−2.56	−2.48	−125.2	−173637.5	−91.95
% with White Collar Jobs	−1.37	−1.40	−1.35	−90.9	−141051	−74.70
% Unemployed	0.09	0.04	0.14	3.6	18260.30	9.67
% ≥HS Education	−6.92	−6.97	−6.87	−287.5	−188649.4	−99.90
% <9^th^ Grade Education	−3.27	−3.33	−3.21	−104	−181656.3	−96.20
% >1 Person per Bedroom Households	0.88	0.83	0.93	33.9	266369.33	141.06

**Figure 2.**
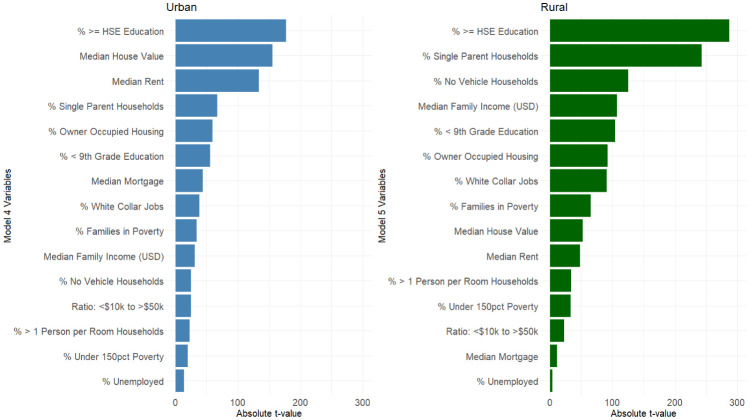
Variable Importance plots for M4 and M5.

Figure 2 shows the ADI component with strongest effect for models 4 and 5 was the percent of people with at least a high school education (t = −177.047; −287.523, respectively). This was statistically significant at p < 0.001, inversely related to COVID-19 prevalence and stronger for rural jurisdictions. The least influential component was also the same, the percent of people unemployed, which was higher and positive for urban jurisdictions (t = 13.331; 3.640). Much of the variable ranking otherwise differed considerably between jurisdictions, with Median House Value, and Median Rent ranking 2^nd^ and 3^rd^ for urban, but only rising to 9^th^ and 10^th^ in rural communities.

## 4. Discussion

Discrepancies between urban and rural counties were evident both in individual SDH measures and their combined effect on prevalence estimates. The differences observed in rank correlation and variable importance appear characteristic of the communities they reflect. Rent and home values tend to be lower in rural jurisdictions, for example, and inversely related to COVID-19 prevalence. In urban areas, fewer residents own private vehicles, thus number of vehicles was less predictive than in rural communities. Generally, stronger associations between ADI components and prevalence were found among rural jurisdictions. Rural models M3 and M5 demonstrated higher deviance explained and M3 had more than twice the change in prevalence per unit ADI compared to urban jurisdiction model M2. Model performance metrics illustrate national rank ADI was more predictive of COVID-19 prevalence in rural communities than urban ones. Together, these results suggest (1) the overall prevalence of COVID-19 is more varied among rural jurisdictions, and (2) the effect of socioeconomic disparity on COVID-19 prevalence is worse for rural jurisdictions over urban ones.

ADI and component measures were instrumental in assessing this contrast between jurisdictions and can aid lawmakers in identifying regions most in need. The health policy implications are (1) that geosocial factors should be considered when identifying communities most at risk of an outbreak, (2) disparate prevalence, morbidity and amenability to interventions can be evaluated for geographic regions and (3) interventions should consider these needs and disparities to adequately control disease spread within a geography. For example, mobile vaccination and testing centers could alleviate limited health access due to low vehicle ownership or poverty.

These results require several qualifications. First, they are time-dependent and reflect an evolving pandemic. Our analysis was limited to the end of August 2020 to inspect the initial phases of disease spread as it relates to geographic characteristics and SDH. Other researchers have found the same general pattern for high COVID-19 incidence in rural communities during the early stages of the pandemic [33]. Temporal modeling with the implementation of various health policy measures and locations may be required to further our understanding of these associations between deprivation and spread but was out of scope for this work.

Second, the granularity of both the classification scheme and level of geography are not ideal for detecting small or nuanced effects. We expect much greater heterogeneity in ADI composites for densely populated regions. Census tract or block group level data may have been more appropriate, but this information for testing results is not currently available nationwide [34]. Third, ADI only captures a handful of SDH that, while widely used, do not account for racial disparities in COVID-19 spread. Race, age and gender should be considered in future modeling efforts for coronavirus prevalence. Asymptomatic spread of the disease likely also undermines our understanding of differential prevalence by county type.

Finally, mortality is a parallel outcome that has substantial weight in policy decisions. Most efforts to understand level of COVID-19 mortality risk are conducted at the patient-level however. Mortality can be evaluated at by geography but is time-lagged, limiting its usefulness in prevention and planning. Another approach might be to use estimates of healthcare access and comorbidities at geographic scales to gauge localized risk of COVID-19 mortality. Such analyses were beyond the scope of this work but remain of interest wherever these data are available.

Additional work is also required to tie in known risk factors and SDH to adequately address long-standing disparities in health outcomes and predict geographies that are most impacted by a pandemic [35]. Rural communities have notably different challenges to access care than those in more densely populated areas [36],[37]. During a pandemic, lack of reliable internet access and transportation may compound the effect of poverty on telehealth services or ambulatory care. Efforts targeting rural communities must navigate these challenges while reducing the disparate burden of poverty [38]. As more data become available on coronavirus cases, we expect finer resolution of geographic data, making it necessary to reevaluate and confirm these findings in smaller community levels.

## 5. Conclusions

Though the majority of COVID-19 cases and deaths occur in metropolitan areas, rural communities continue to struggle with highly disparate health outcomes and in some jurisdictions, higher per capita COVID-19 prevalence. The reasons for this geographic difference in prevalence are many but an abundance of research implicates rural health disparity, here measured as an index of deprivation, as exacerbating the pandemic. The underlying economic and practical burdens these communities face have influenced access to care and effective policy to combat the virus likely will need to address these concerns.
